# Biodiesel from *Citrullus colocynthis* Oil: Sulfonic-Ionic Liquid-Catalyzed Esterification of a Two-Step Process

**DOI:** 10.1155/2014/540765

**Published:** 2014-05-29

**Authors:** Yasir Ali Elsheikh, Faheem Hassan Akhtar

**Affiliations:** Department of Chemical and Materials Engineering, King Abdulaziz University, P.O. Box 344, Rabigh, Saudi Arabia

## Abstract

Biodiesel was prepared from *Citrullus colocynthis* oil (CCO) via a two-step process. The first esterification step was explored in two ionic liquids (ILs) with 1,3-disulfonic acid imidazolium hydrogen sulfate (DSIMHSO_4_) and 3-methyl-1-sulfonic acid imidazolium hydrogen sulfate (MSIMHSO_4_). Both ILs appeared to be good candidates to replace hazardous acidic catalyst due to their exceptional properties. However, the two sulfonic chains existing in DSIMHSO_4_ were found to increase the acidity to the IL than the single sulfonic chain in MSIMHSO_4_. Based on the results, 3.6 wt% of DSIMHSO_4_, methanol/CCO molar ratio of 12 : 1, and 150°C offered a final FFA conversion of 95.4% within 105 min. A 98.2% was produced via second KOH-catalyzed step in 1.0%, 6 : 1 molar ratio, 600 rpm, and 60°C for 50 min. This new two-step catalyzed process could solve the corrosion and environmental problems associated with the current acidic catalysts.

## 1. Introduction

The majority of the world energy needs are supplied through petroleum resources. The increase in energy demand and pollution problems caused by industrialization has urged researchers and economists to find new sources of energy. One of the feasible energy sources is the use of plant oils, which is readily available and environmentally acceptable [1].

Biodiesel is an alternative diesel fuel that consists of alkyl monoesters of fatty acids from vegetable oils or animal fats. Pure vegetable oils have been used in the past in diesel engine. However, there have been many problems linked with the direct use of vegetable oils (VOs) in the diesel engine, such as high viscosities and the lower volatilities [2].

The competition between edible oil and fuel needs may cause worldwide disproportion in the food industry and market demands. Moreover, using all edible oils for biodiesel production will not be enough for worldwide fuel demand [3, 4]. Therefore, there is a need for nonedible and inexpensive feedstock such as* Citrullus colocynthis* oil for biodiesel production.* Citrullus colocynthis* is a desert plant of the Cucurbitaceae family. It grows in many countries with semidry lands and its seeds are rich in oil which can encourage their sustainable agriculture to produce an economic biodiesel with a competitive price [5].

Transesterification is one of the accepted processes for the production of biodiesel from oils and fats with alcohols in the presence of homogeneous catalysts [6] or heterogeneous catalysts [4, 7]. The homogeneous catalysts have been proven to be more practical in application [8]. The alkaline catalyst is capable of producing higher yield and purity of biodiesel with reaction time of between 30 and 60 min [6] and limits the FFA content not more than 1.0% [9, 10]. This has made it only suitable for the processed or refined vegetable oil to be used as the feed material due to its low FFA and purer triglycerides (TG) content compared to the crude vegetable oil [11].

During transesterification of crude oils, FFA can easily react with an alkali catalyst producing soaps and water and, consequently, the downstream recovery and purification of the product becomes difficult [6, 12]. In this regard, several researchers have reported that for the use of alkali catalyst, the oil should have lower FFA content (<1.0%) [13, 14]. Therefore, acid catalyzed processes are preferred. Unfortunately, acid catalyzed processes require relatively high amounts of alcohol and pressure, which necessitates an increase in the size of the reactor, extensive conditioning, and purification steps to recover unreacted alcohol, as well as the catalyst, from the reaction products. Furthermore, the use of excessive alcohol complicates the recovery of glycerol due to its high solubility in alcohol [15, 16]. Another negative feature of the conventional catalyzed process is that the catalysts are not reusable [17]. All these drawbacks will be adding more cost to the production expense and affecting the biodiesel price.

Canakci and Gerpen have developed a new combined catalytic process for handling different feedstocks with high FFA [15]. The process involves conducting the acid catalyzed esterification in the first step for the purpose of lowering the FFA content to an accepted range followed by the addition of alkaline catalyst, after the removal of the acid catalyst, to complete the transesterification process. Nevertheless, this process too has drawbacks where acidic waste water is produced along with alkyl esters from the esterification reaction. The problem of managing the highly acidic effluent, the difficulty in catalyst recovery processes, and the high cost of stainless steel equipment needed for the acidic reaction media became the main limitations for applying this process [13].

More recently, in our previous work [18], we studied 1-butyl-3-methylimidazolium hydrogen sulfate acidic IL as precatalyst for esterification of crude palm oil. We also introduced KOH as second-step catalyst. Soon, in other work, we used triethylammonium hydrogen sulfate based IL as the pre-catalyst in the first stage, in which the acid value of crude palm oil was reduced from 6.98 to 1.24 mg KOH/g of oil followed by the use of alkali-catalyzed transesterification in the second stage [19].

The use of IL as a catalyst has the prospect to develop the economic and environmental aspects of biodiesel preparation, due to their properties of less corrosion effects, separable, recyclable, direct, and continuous processing and less waste water formation. Moreover, ILs catalysis would decrease the number of reactions and purification steps required in the biodiesel preparation and separation, thus allowing for more competitive economical processing and yielding higher purity of esters [20]. The ionic liquids also have negligible vapor pressure which avoids costly pressure control apparatus and have good thermal transport properties [21, 22].

**Figure 1:**
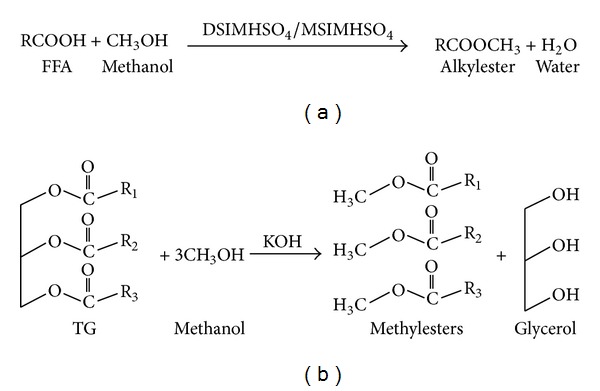
Preparation of CCOME via two-step catalyzed scheme. (a) Esterification of FFA with methanol to form alkyl ester and water; (b) transesterification reaction of TG with methanol to form methyl esters and glycerol. R_1_, R_2_, and R_3_ are alkyl groups.

In this work, to study the activity of another route, cation chains in imidazolium, DSIMHSO_4_, and MSIMHSO_4_ were synthesized and used as catalysts for esterification of CCO with FFA content of 3.18% (equivalent to 6.36 mg KOH/g oil). As illustrated in Figure 1, a two-step catalytic process has been applied to produce biodiesel from CCO. Firstly, the FFA in the CCO was esterified with methanol in the presence of one of the functionalized ILs. At the second step, KOH was introduced to catalyze the transesterification of the esterified CCO with methanol.

## 2. Materials and Methods

### 2.1. Materials

The chemicals purchased from Sigma-Aldrich, Fluka, or Merck companies include anhydrous methanol (>99.8%), 1-methylimidazole (>99%), sulfuric acid (97%), imidazole (>99.8%), chlorosulfonic acid (99.0%), dichloromethane (>99.8%), n-hexane (>99%), KOH (analytical grades), ethyl acetate, and diethyl ether. All the fatty acid methyl esters reference standards used for GC characterization were of chromatographic purity (>99%). All chemicals were used without drying or any further purification.

### 2.2. Oil Extraction

Bitter apple seeds were obtained from Atbara local market-Sudan. The crushed seeds (125 g) were introduced to a soxhlet extraction system fitted with a 500 mL three-necked round bottom flask and a condenser. The extraction was employed, according to the AOAC official method of 963.15, on a water bath using 200 mL of n-hexane as extracting solvent [23]. After 8 hours, the solvent was recovered under vacuum at 45°C. The oil gained was 48 wt% (weight of oil/weight of seeds).

### 2.3. Pretreatment of CCO

Since water and solid particles presented in oil create problems during transesterification [24], CCO was pretreated before the reaction by heating above 100°C for 1 h [25]. Solid particles were removed using a centrifuge and water was removed by mixing CCO with 10 wt% silica gel followed by stirring the mixture and vacuum filtration using Whatman filter paper for the removal of silica gel [26]. This step was performed three times to ensure complete removal of the water present in the oil.

### 2.4. Characterization of CCO

The physicochemical properties of the CCO were determined and shown in Table 1. The CCO density was measured with an Anton Paar DMA5000 instrument, while the kinematic viscosity was determined using an Ubbelohde glass viscometer. The fatty acid composition, shown in Table 2, was determined using the official and practice method AOCS (Ce 1–62) using Agilent Hewlett-Packard 6890 series gas chromatograph equipped with flame ionization detector, SP-2340 capillary column (60 m in length, 25 mm of internal diameter, and 0.2 mL film thickness), and a split ratio of 100 : 1. CCO was found to be holding primarily eleven fatty acids. Of these, linoleic (9,12-octadecadienoic) acid, palmitic (hexadecanoic) acid, stearic (octadecanoic) acid, oleic (octadec-9-enoic) acid are frequently presented in most of oils. The other fatty acids, including myristic, palmitoleic, margaric, linolenic, arachidic, gadoleic, and behenic were present in minor amounts.

The oil had a significantly high content of FFA (3.18%) evaluated by AOCS standard titration method, which is higher than the content (>1.0%) permitted in oil to be catalyzed by alkali catalysts.

### 2.5. Preparation of IL Catalysts

#### 2.5.1. Synthesis of DSIMHSO_4_

**Figure 2:**
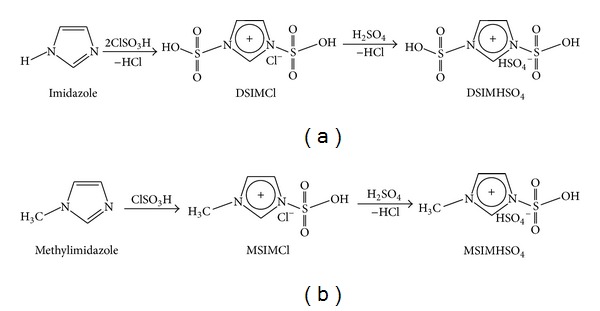
Synthesis of ILs via two-step scheme: (a) synthesis of DSIMHSO_4_ by direct metathesis between DSIMCl and H_2_SO_4_; (b) synthesis of MSIMHSO_4_ by direct metathesis between MSIMCl and H_2_SO_4_.

DSIMHSO_4_ was synthesized in two steps as illustrated in Figure 2(a). Chlorosulfonic acid (0.2 mol, 23.3 g) and imidazole (0.1 mol, 6.8 g) were stirred with 50 mL dichloromethane in a three-necked flask in ice bath for 18 h under pressure of N_2_ gas. The resultant mixture was separated by decantation and the viscous yellow 1,3-disulfonic acid imidazolium chloride (DSIMCl) was collected, after being washed repeatedly with ethyl acetate. Then under vigorous stirring, a stoichiometric amount of concentrated H_2_SO_4_ was added drop wise over a period of 15 min at 0°C. After the dropping was finished, the reaction mixture was slowly heated up to 40°C for 6 h. The resultant viscous yellow liquid was washed with 50 mL dichloromethane and then vacuum-dried at 70°C for 12 h, resulting in DSIMHSO_4_ with purity of >98%.

#### 2.5.2. Synthesis of MSIMHSO_4_

^1^MSIMHSO_4_ as demonstrated in Figure 2(b) was synthesized with purity of >99% by direct metathesis between 3-methyl-1-sulfonic acid imidazolium chloride (MSIMCl) and H_2_SO_4_ via the same method mentioned above.

### 2.6. Recognition of ILs

#### 2.6.1. Characterization

HNMR spectroscopy was used to confirm the structures of synthesized ILs and for this ^1^HNMR (Bruker Avance 300 MHZ) spectroscopy in D_2_O was used, while a CHNS-932 (LECO) apparatus was used for the elemental analysis.

#### 2.6.2. ILs Decomposition and Melting Point Measurements

A PerkinElmer Pyris II differential scanning calorimetry (DSC) instrument was used to detect the thermal behavior of the prepared ILs samples following the procedure described by Song et al., in aluminum sealed pan [27]. The sample of ≤10 mg was firstly cooled to −60°C from ambient temperature and then scanned, on heating rate of 10°C/min, to 150°C under a flow of nitrogen.

For investigating the thermal stability of the prepared ILs, thermogravimetric measurements were conducted using PerkinElmer TGA, Pyris I. A sample of ≤10 mg holds in a capped Al pan at a heating rate of 10°C/min from 50°C to 600°C under nitrogen flow [28].

#### 2.6.3. Viscosity and Density Measurements

The absolute viscosity of the ionic liquids was measured with the use of Ubbelohde type glass capillary viscometer. The value of the viscosity was measured with an accuracy of 5% from the measurements of viscosity between the temperatures 20°C and 80°C.

The density of the ionic liquids was measured by weighing a constant volume of ionic liquid between temperatures 20°C and 80°C.

### 2.7. Two-Step Catalytic Process

#### 2.7.1. IL-Catalyzed Esterification of CCO

The objective of this step was to reduce the FFA contents of CCO. Before starting the esterification reaction, the CCO was firstly preheated to the designated temperatures (100–170°C) on a heating plate and under reflux condition. Under agitation, a fixed amount of freshly prepared methanol-IL solutions was added to the oil and the reaction continued to the established times.

Firstly, in order to evaluate their catalytic activity a concentration range of 3.0–5.0 (wt%) of MSIMHSO_4_ or DSIMHSO_4_ was explored for esterifying the CCO under different reaction conditions. Then using the best catalyst, the parameters affecting the esterification reaction were investigated and optimized. These parameters included methanol/CCO molar ratio range from 3 : 1 to 21 : 1 and 8 levels of temperature (100–170°C). A rate of stirring of 600 rpm was applied for all experiments, which is similar to the mixing rate approved by Vicente et al. to be sufficient to overcome the mass transfer limitation during biodiesel production process [29].

After the proposed time, 30, 45–180 min, the product formed was washed with diethyl ether and the different layers were separated carefully. Following the removal of the IL layer, the layer containing the treated CCO was subjected to alkali catalyzed hydrolysis.

In order to confirm the conversion of FFA during the process, the samplings were done manually and the acid value was calculated from the acidity reduction using

(1) Acid  Value(mg KOHg) =(Volume  of  NaOH  used×Molar  mass×NaOH  normality)  ×(CCO weight(g))−1.

The acid value was then used to calculate FFA content as shown in the following [18]:

(2) $$\begin{array}{c} \text{FFA}\;\text{conversion}=\left(\frac{\text{A}{\text{V}}_{i}-\text{A}{\text{V}}_{t}}{\text{A}{\text{V}}_{i}}\right)\times 100, \end{array}$$

where AV_*i*_ is initial acid value of the mixture and AV_*t*_ is the acid value at a “*t*” time.

#### 2.7.2. KOH-Catalyzed Transesterification of CCO

This methodology has been previously described in our work for transesterifying the esterified oil using 1.0 wt% KOH, a temperature of 60°C, 600 rpm with a 6 : 1 molar ratio of methanol to CCO for 50 min reaction time [18].

### 2.8. Recovery of ILs

On achievement of esterification reaction, the remaining alcohol was distilled under vacuum. CCO and IL were immiscible layers; therefore the separation of lower layer IL from CCO was simple. IL was centrifuged, washed thoroughly with ethyl acetate, and then vacuum dried at 760 mmHg and 50°C for 3 h.

### 2.9. Analysis of Biodiesel

The* Citrullus colocynthis* oil methyl esters (CCOME) were analyzed in duplicate using a Shimadzu Gas Chromatograph (GC-2010) equipped with AOC-20i automatic injection and a flame ionization detector (FID). The capillary column was a SP-2330 with a length of 25 m, a film thickness of 0.5 *μ*m and an internal diameter of 0.32 mm. The GC oven was kept at 150°C for 5 min, heated at 3°C/min up to 210°C, and a total analytical time was 29 min. The carrier gas was helium (0.7 mL/min). Helium (purity ≥99.95 mol%) was used as carrier gas, at a flow rate of 0.8 mL/min and split ratio of 100 : 1 and also used as an auxiliary gas for the FID. The injection port temperature was equal to the detector temperature (250°C). 10 *μ*L of each sample was injected automatically after being dissolved into n-hexane. Methyl heptadecanoate was employed as internal standard.

The esters content for each experiment was calculated from the contents of CCOME in the ester phase as investigated by GC and the theoretical yield calculated according to [30]

(3) $$\begin{array}{c} \text{Esters}\;\text{content}\left(\%\right)=\frac{\text{Actual}\;\text{esters}\;\text{conc}.}{\text{Theoratical}\;\text{esters}\;\text{conc}.}\times 100. \end{array}$$

The actual concentration is obtained from GC, while the theoretical concentration is calculated from the stoichiometric reaction.

The methyl esters yield was calculated by means of the following formula [31]:

(4) Yield  of  methyl  esters(%)  =grams  of  methyl  esters  producedgrams  of  CCO  used×100.

## 3. Results and Discussion

### 3.1. Recognition of Ionic Liquids

The results obtained from the NMR spectroscopy of the synthesized ILs as given below confirmed the structure of respective ILs.

MSIMHSO_4_: the NMR spectroscopic data recorded is ^1^HNMR (400 MHz, DMSO): *δ* = 3.756 (s, 3H, N–CH_3_), 7.389 (s, 1H, N–C–CH–C), 7.554 (s, 1H, N–CH–C–N), 8.825 (s, 1H, N–CH–N), 11.86 (s, 1H, O–H), and 14.242 (s, 1H, N–SO_3_–H).

DSIMHSO_4_: the NMR spectroscopic data recorded is: ^1^HNMR (400 MHz, DMSO): *δ* = 7.476 (s, 2H, N–C–CH–C and N–CH–C–N), 8.739 (s, 1H, N–CH–N), 10.984 (s, 1H, O–H), and 13.852 (s, 2H, N–SO_3_–H).

The observation of ^1^HNMR analysis of DSIMHSO_4_ showed dissimilarities to MSIMHSO_4_. This is due to the DSIMHSO_4_ structure having two symmetrically side chains of acidic sulfonated groups resulting in 4 peaks compared to 6 peaks in MSIMHSO_4_. However, in these spectra, the peaks related to the proton in N–C–CH–C, N–CH–C–N, OH in the HSO_4_, and OH in the SO_3_H groups of DSIMHSO_4_ and MSIMHSO_4_ were shown great similarities in positions.

### 3.2. Melting Point

**Figure 3:**
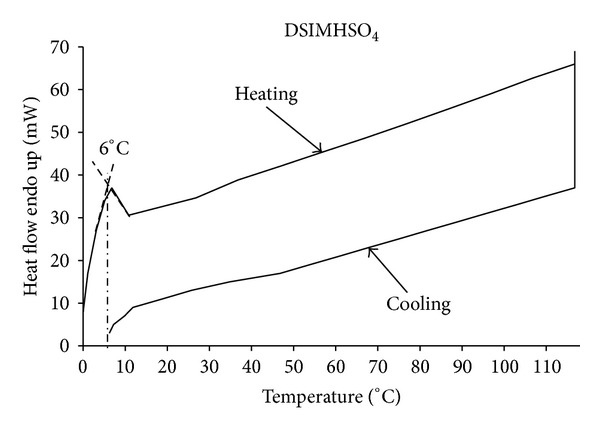
DSC curve for melting point determination of DSIMHSO_4_.

**Figure 4:**
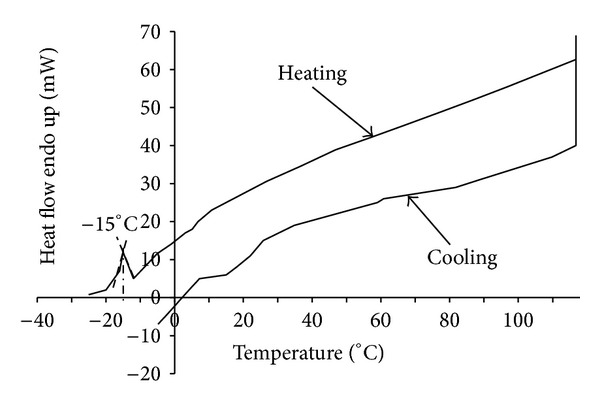
DSC curve for melting point determination of MSIMHSO_4_.

The synthesized ionic liquids, DSIMHSO_4_ and MSIMHSO_4_, showed melting temperatures at 6 ± 0.2°C and −15 ± 0.2°C, respectively, presented in Figures 3 and 4. Since both the ionic liquids were molten at degrees below room temperature, hence they are considered as room temperature ionic liquids (RTILs).

### 3.3. Decomposition of ILs

Both of the designed ionic liquids, DSIMHSO_4_ and MSIMHSO_4, _were tested in order to determine the thermal decomposition (*T*_dec_, °C) in the range of 50–550°C. The aim of this test was to understand their behaviour during esterification at high temperatures.

**Figure 5:**
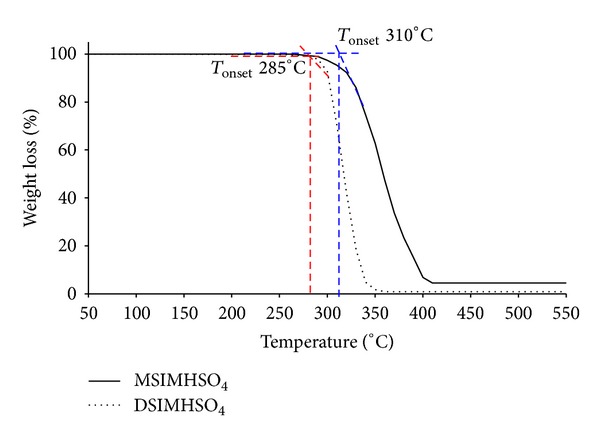
Thermogravimetric analysis (TGA) curves of the thermal decomposition of DSIMHSO_4_ and MSIMHSO_4_.

Figure 5 presents the TGA curve of single stage decomposition consisting of two characteristic temperatures for both ILs. It is clear from the figure that DSIMHSO_4_ is less stable as the onset temperature of mass change found at* c.* 285°C, whereas for MSIMHSO_4_ this was at* c.* 310°C. Final decomposition of MSIMHSO_4_ appeared to be completed at* c.* 420°C with the analogs at* c.* 349°C. Although the thermal decomposition of MSIMHSO_4_ proceeds over a wider temperature range than DSIMHSO_4_, their maximum rate of weight loss is almost the same.

### 3.4. Density and Viscosity

**Figure 6:**
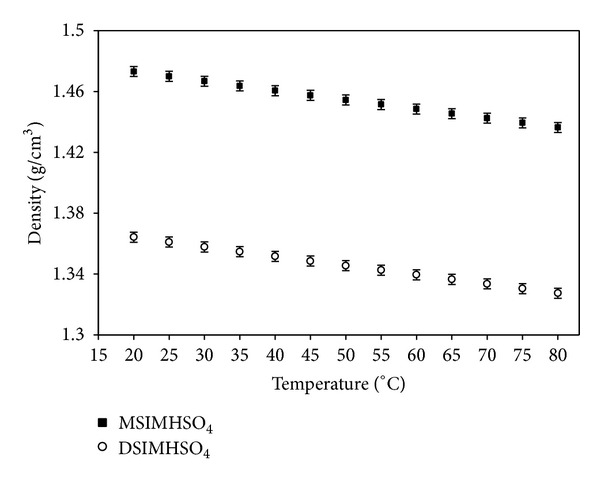
Effect of temperature on density of DSIMHSO_4_ and MSIMHSO_4_.

The present experimental values of absolute density for both the ionic liquids as a function of temperature are presented in Figure 6. The densities of the investigated ionic liquids decrease linearly as the temperature increases. It is observed that the density decreases as the length of alkyl chain of the cation increases.

**Figure 7:**
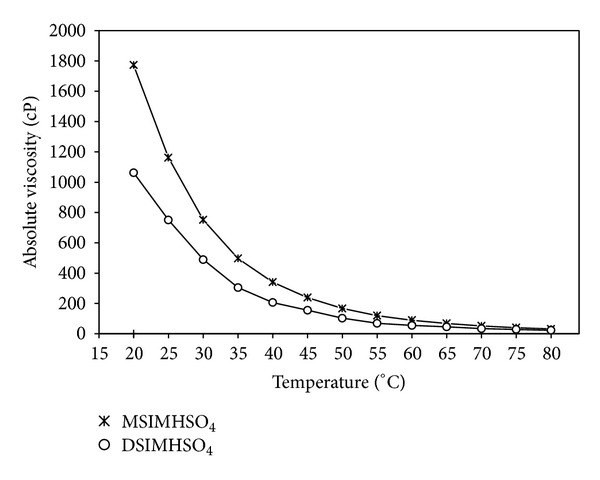
Effect of temperature on absolute viscosity of DSIMHSO_4_ and MSIMHSO_4_.

The determined viscosities of the ionic liquids as a function of temperature are shown in Figure 7. The viscosity decreases as the temperature increases. The viscosity of both the ionic liquids approaches to zero at the maximum temperature of 80°C.

### 3.5. Optimization of the First Stage Using IL as Catalyst

#### 3.5.1. Effect of Catalyst Type and Concentration on FFA Conversion

**Figure 8:**
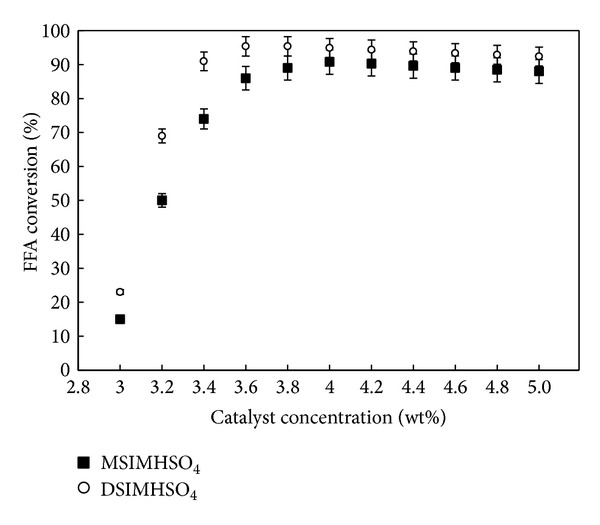
FFA conversion yield versus IL concentration. The reaction conditions were 12 : 1 methanol/CCO molar ratio, 150°C, and 600 rpm for 3 h.

Catalyst type is one of the main factors affecting the rate reaction. The performances of DSIMHSO_4_ and MSIMHSO_4_ in the concentration range of 3.0–5.0 wt% are presented in Figure 8.

Keeping other parameters constant such as 150°C reaction temperature, 600 rpm agitation rate, excess molar ratio of methanol to CCO of 12 : 1, and 3 h reaction time, it was observed that with the increase of the catalyst amount, the FFA conversion increases rapidly. The lower catalytic concentration of 3.0% of both ILs was insignificant to guide the reaction for completion. With DSIMHSO_4_ catalytic system, it was seen that the FFA conversion rate increased as its concentration was increased from 3.2 wt% to 3.6 wt% to reach a conversion of 95.4%. While with the other catalyst (MSIMHSO_4_), a 4.0 wt% was required to achieve its highest conversion of 90.8%. Therefore, DSIMHSO_4_ was selected for further investigations as it was found to be superior to MSIMHSO_4_. This may be due to that the imidazolium is acting as an unstable base cation [32]. Once this unstable base cation was combined with the single acidic sulfonic chain in MSIMHSO_4_, it might have acted as a weak acidic IL. When both nitrogens were occupied with two acidic sulfonic chains to give DSIMHSO_4_, this perhaps revealed a stronger proton donor IL than MSIMHSO_4_.

Further increase in catalyst concentration neither enhances further conversion nor raises the ester content. In fact, with further increase in the concentration of both catalysts, beyond 4.2 wt%, there were dramatic decreases in the conversion rates. This consequence is in agreement with our previous finding [18] detailed in a process of esterifying crude palm oil and alcohol with Brønsted imidazolium ILs.

This action was perhaps due to that increasing the IL concentration increases the viscosity of the reaction media and, as a result, decreases the contact between the two reactants throughout the esterification.

#### 3.5.2. Effect of Molar Ratio on FFA Conversion

**Figure 9:**
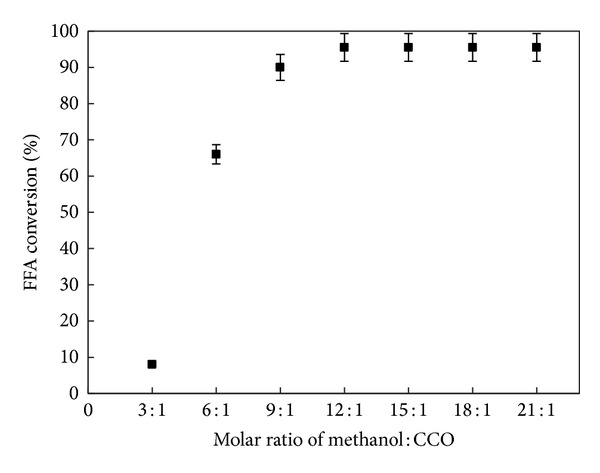
FFA conversion yield versus methanol/CCO molar ratio. The reaction conditions were 3.6** **wt% DSIMHSO_4_, 150°C, and 600 rpm for 3 h.

The variation of the FFA conversion with increasing the methanol-CCO ratios from 3 : 1 to 21 : 1 was shown in Figure 9. In this section, the DSIMHSO_4_ concentration as optimized in the previous section was recognized. Experiments were carried out at fixed conditions of 150°C, and agitation rate of 600 rpm for 3 h. It was observed that the FFA conversion was rapidly improved with increasing the molar ratio. The molar ratio 6 : 1 was commonly used with alkali-catalyzed transesterification; however, acid-catalyzed esterification process requires an excess of alcohol [17]. With the lower molar ratio (3 : 1) the conversion was found as low as 7.8%. But, when the molar ratio increased to 6 : 1 the conversion increased to 67.6%. Consequently, it is not beneficial to acidify the CCO to below the maximum accepted acid value of 2.0 mg KOH/g oil (equivalent to >70% FFA conversion of our CCO), as recommended by Freedman et al. [13] for the oil to undergo alkali-catalyzed transesterification.

When higher molar ratios were applied (≥9 : 1), the reaction was improved leading to high FFA conversion within 3 h min using 3.6 wt% of DSIMHSO_4_ at 170°C. However, when the molar ratio exceeded 12 : 1, the rate conversion became independent of composition. Accordingly, the ratio 12 : 1 appeared optimum for achieving maximum conversion. This consequence is consistent with our previous studies [18, 22] resulting in 12 : 1 as an optimal molar ratio of alcohol to oil.

#### 3.5.3. Effect of Temperature on FFA Conversion

**Figure 10:**
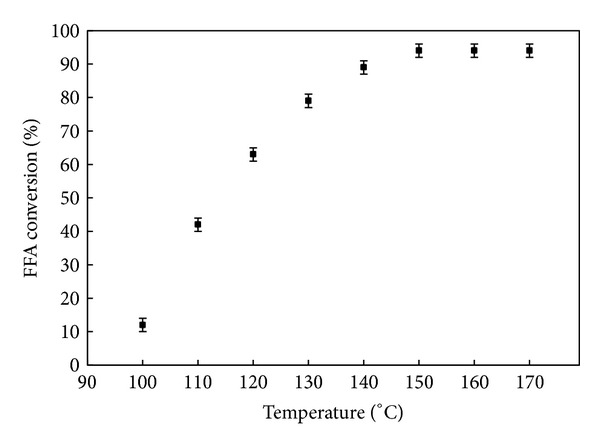
FFA conversion yield versus reaction temperature. The reaction conditions were 12** **:** **1 methanol/CCO molar ratio, 3.6** **wt% DSIMHSO_4_, and 600** **rpm for 3** **h.

The influence of temperature on esterification was studied using the fixed conditions of 12 : 1 alcohol/CCO ratio and a catalyst concentration of 3.6 wt% for 3 h. As expected, the acidity, contrasting to the FFA rate of reaction, was reduced with increasing the temperature from 100 to 170°C. As can be seen in Figure 10, the highest FFA conversions, lowest acidity as well, were obtained at the highest temperatures of 140°C, 150°C, 160°C, and 170°C, giving conversions of 89.2, 95.4, 95.5, and 95.6%, respectively. Also, at 130°C, CCO was reduced to below the recommended value leading to a conversion of 79%.

The FFA conversion was found to be highly dependent on the reaction temperature as the conversion rate increased with increasing the temperature, indicating that it is an endothermic reaction. Even though the highest reaction rate was achieved at 170°C, the temperature 150°C was selected as the optimum temperature in our work for esterifying the CCO using DSIMHSO_4_ as catalyst as it required less condensation stages for cooling, less power, and shorter time for cooling the product.

#### 3.5.4. Effect of Reaction Time on FFA Conversion

**Figure 11:**
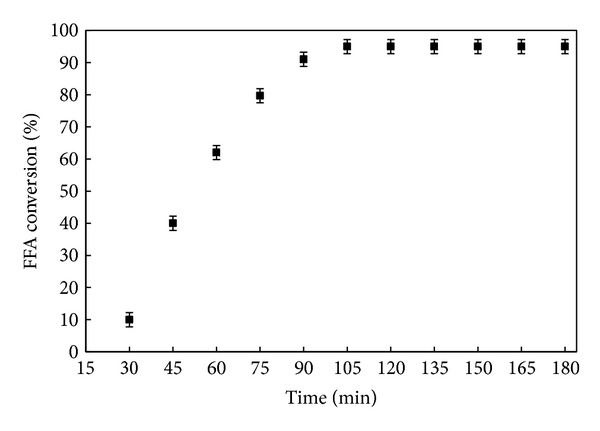
Conversion of FFA versus reaction time. The reaction conditions were 12** **:** **1 methanol/CCO molar ratio, 3.6** **wt% DSIMHSO_4_, 150°C, and 600** **rpm.

The results are displayed in Figure 11 and showed the variation of optimal parameters versus time. In this part, to establish the effect of reaction time on FFA conversion, experiments were carried out at different times ranging from 30 to 180 min in the presence of 3.6 wt% DSIMHSO_4_ catalyst at a reaction temperature 150°C, methanol/CCO molar ratio of 12 : 1, and agitation rate of 600 rpm.

The results showed that the FFA conversion increased with time. Over 60% of FFA was converted into CCOME within 1 h, while the highest yields were achieved after 75 min. It was also observed that increasing the reaction time beyond 105 minutes does not have much effect on reducing the acid value as preferred. This may perhaps be due to the negative effect of water produced during the esterification of FFA, which stopped further reaction.

### 3.6. KOH-Catalyzed Transesterification (Second Step)

**Figure 12:**
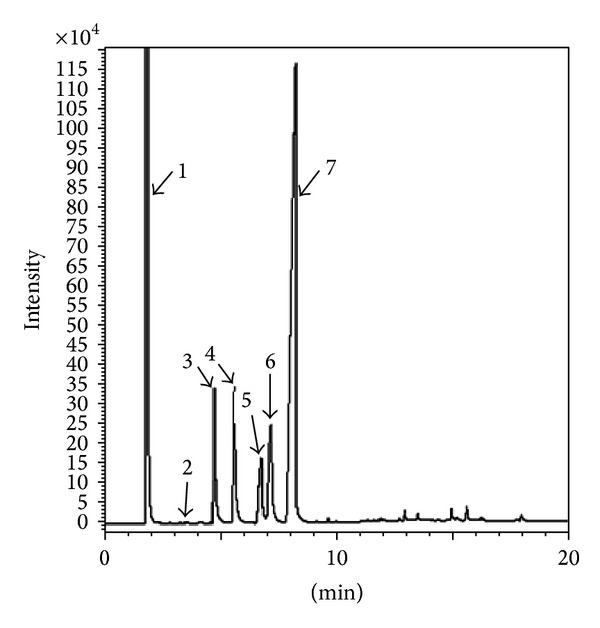
GC chromatogram of the produced CCOME. The peaks 1–7 showed n-hexane (solvent), methyl myristate, methyl palmitate, methyl heptadecanoate (internal standard), methyl stearate, methyl oleate, and methyl linoleate, respectively.

Under the operational conditions that have been mentioned, the transesterification was carried out using KOH, which resulted in the favored products, CCOME and glycerol. In this work, the CCO lowest acid value pretreated by the DSIMHSO_4_ was 0.29 ± 0.021 mg KOH/g oil, which is below the recommended acidity to be <2.0 mg KOH/g oil for alkali catalysis [9, 13, 33]. The well dried upper layer, after decanting the lower KOH glycerol layer and washing with water, produces biodiesel purity of 98.2% (see Figure 12) using the optimal conditions of KOH 1.0 wt%, methanol to CCO mole ratio of 6 : 1, and 60°C reaction temperature for 50 min.

### 3.7. Characterization of CCOME

The biodiesel standards have been established to ensure its commercialization with an accepted fuel quality [19]. The selected fuel properties of the CCOME, presented in Table 3, were studied according to the approved testing procedures. The results obtained, when compared with the specifications of the American Standards for Testing Material (ASTM D6751) biodiesel standards, were guaranteed good parameters within the accepted ranges.

### 3.8. Ester Content

**Figure 13:**
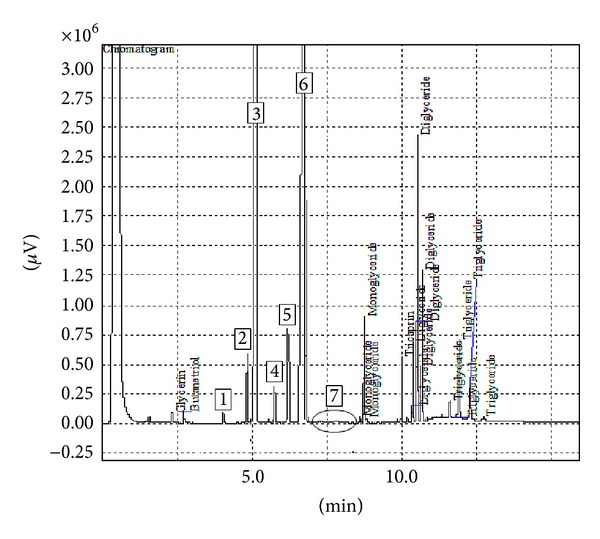
Methyl ester content in CCO.

The methyl esters content in the CCO biodiesel sample was determined by GC and measured by comparing their retention times with peak areas of standard sample. As can be seen in Figure 13 and Table 4, the most abundant methyl esters in CCO biodiesel sample is methyl myristate (3.83 min), followed by methyl palmitate (4.35 min), methyl heptadecanoate standard (5.12 min), methyl stearate (5.65 min), methyl oleate (6.15 min), and methyl linoleate (6.68 min). The others were detected in the retention time range of 6.87–8.30 min which may possibly contain small amounts of minor linolenate, arachidate, gadoleate, and/or behenicate methyl ester. The content of methyl linoleate was distinguished between methyl esters with the highest composition (63.75 ± 0.47%).

### 3.9. Reusability of Ionic Liquid

DSIMHSO_4_ was recovered by separation after the completion of esterification process. DSIMHSO_4_ was dried under vacuum at 80°C for 4 h to remove the traces of water before the use of IL for each recycle.

**Figure 14:**
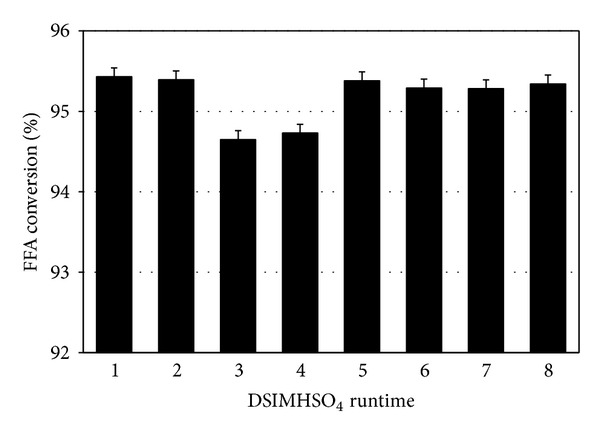
Reusability of DSIMHSO_4_.

The experimental results revealed that the recovery of IL was above 95% and without any weight losses. This is because the ILs showed no miscibility with the produced esters. As illustrated in Figure 14, DSIMHSO_4_ was recycled 8 times with almost the same FFA conversion.

**Figure 15:**
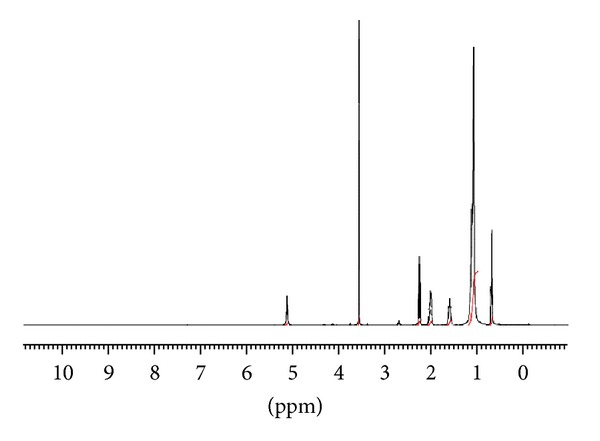
Proton NMR spectrum for CCOME content. A conversion of 95.4% obtained after 105 min at 150°C, 3.6 wt% of recycled DSIMHSO_4_, and 12 : 1 molar ratio of methanol/CCO.

**Figure 16:**
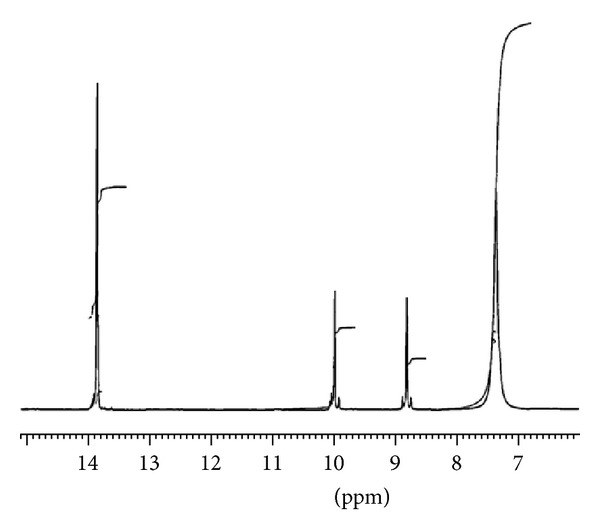
Recovered DSIMHSO_4_ after the 8th run for esterification reaction.

After the last run of the transesterification reactions, the CCOME sample and the recovered DSIMHSO_4_ were investigated by ^1^H NMR to check the purity of biodiesel, that is, no traces of IL left, and to check the efficiency of the method used for purifying the IL. Figures 15 and 16 presented the proton NMR results for the biodiesel achieved after the 8th run and the DSIMHSO_4_ after it has been recovered after the same run, respectively.

As can be seen from the CCOME spectrum, the biodiesel is pure and no traces of IL were presented. The data detailed in Figure 15 for the proton chemical shifts were analyzed according to the details available elsewhere [34, 35]. The singlet sharp peak of methoxy proton was observed at 3.55 ppm. The *α*-CH_2_ (O–CO–CH_2_–) to the ester group is at 2.25 ppm and the *β*-CH_2_ (O–CO–C–CH_2_–) to the ester group is at 1.6 ppm. The three peaks are the distinct peaks of methyl esters presence in the biodiesel sample. Other detected peaks were triplet at 0.8 ppm of terminal methyl proton, a large signal at 1.16 ppm was observed clearly which was assigned for methylene protons of carbon chain; a signal at 5.14 ppm is related the ^1^H located at the olefinic methyl esters [34].

The absence of peaks between 4.0 and 4.3 ppm suggested that no glycerol traces were present in the biodiesel sample. Moreover, the absence of DSIMHSO_4_ peaks in Figure 15 indicated that no DSIMHSO_4_ remained in the CCOME after the 8th run.

Therefore, it is believed that the selected IL can be considered as a potential replacement for the conventional catalyst being used.

## 4. Conclusion

Both ILs showed good catalytic activity for the esterification of FFA and reducing the CCO acid value. In comparison a disulfonic chain of DSIMHSO_4_ was compared with single sulfonic chain in MSIMHSO_4_. Compared to homogeneous acidic catalysts, ionic liquids in general have the prospect to reduce environmental impact usually associated with chemical processes due to their ease of recoverability and reusability, which ensures less waste water generation. Less corrosion effect is another advantage which makes ILs more economical and favorable since this can guarantee continuous processing.

## Figures and Tables

**Table 1:** Physical and chemical properties of CCO.

Property	Standard method	CCO
Density, 20°C (kg/m^3^)	ASTM D4052	925.7
Kinematic viscosity, 38°C (mm^2^/s)	ASTM D445	35.20
Acid value (mg KOH/g oil)	ASTM 664	2.30
Iodine value (g I/100 g oil)	AOCS Cd1-25	107.4
Flash point (K)	ASTM D93	509
Pour point (K)	ASTM D97	285
Cloud point (K)	ASTM D2500	279
Carbon residue (wt.%)	ASTM D4530	0.27
Cetane number	ASTM D613	37.4
High heating value (MJ/kg)	ASTM D240	39.56
Sulfur content (wt.%)	ASTM D5453	0.009
Ash content (wt.%)	ASTM D482-91	0.009
Distillation temp. (°C), 95%	ASTM D86	368

**Table 2:** Fatty acid composition (wt.%) of CCO.

Acids	wt.%
Myristic acid (C14:0)	0.70
Palmitic acid (C16:0)	10.53
Palmitoleic acid (C16:1)	0.05
Margaric acid (C17:0)	0.14
Stearic acid (C18:0)	9.57
Oleic acid (C18:1)	14.07
Linoleic acid (C18:2)	64.65
Linolenic acid (C18:3)	0.10
Arachidic acid (C20:0)	0.12
Gadoleic acid (C20:1)	0.06
Behenic acid (C22:0)	0.01

**Table 3:** Fuel parameters of CCOME as compared to ASTM standards.

Property	Unit	Test method	CCOME in this work	ASTM D 6751-02
Density, 15°C	kg/m^3^	ASTM D4052	875.7	870–900
Kinematic viscosity, 40°C	mm^2^/s	ASTM D445	4.486	1.9–6.0
Flash point	°C	ASTM D93	174	130 min.
Specific gravity, 15°C	—	ASTM D4052	0.889	0.88–0.90
Iodine value	g I_2_/100 g oil	AOCS Cd1-25	111.46	120 max
Distillation temperature, 95%	°C	ASTM D86	341	360 max.
Cetane number	wt.%	ASTM D613	62.4	47 min
Water content	wt.%	ASTM D6304	0.02	0.05 max.
Acid value	mg KOH/gm	ASTM D664	0.29	0.80 max.
Ester content	wt.%	—	98.2	—

**Table 4:** Methyl esters composition (wt.%) of CCO biodiesel.

Methyl ester	Equivalent chain length	CCO biodiesel
Myristate	C14:0	0.60 ± 0.06
Palmitate	C16:0	10.14 ± 0.20
Stearate	C18:0	9.13 ± 0.14
Oleate	C18:1	13.78 ± 0.23
Linoleate	C18:2	63.75 ± 0.47
Others	0.10	0.15 ± 0.02
